# Incidence of Acute Kidney Injury Is Lower in High-Risk Patients Undergoing Percutaneous Coronary Intervention Supported with Impella Compared to ECMO

**DOI:** 10.1007/s12265-021-10141-9

**Published:** 2021-07-29

**Authors:** Julian Schweitzer, Patrick Horn, Fabian Voss, Milena Kivel, Georg Wolff, Christian Jung, Tobias Zeus, Malte Kelm, Ralf Westenfeld

**Affiliations:** 1 Division of Cardiology, Pulmonology and Vascular Medicine, Heinrich Heine University Düsseldorf, Medical Faculty, Moorenstrasse 5, 40225 Düsseldorf, Germany; 2 CARID (Cardiovascular Research Institute Düsseldorf), Heinrich Heine University Düsseldorf, Medical Faculty, Moorenstrasse 5, 40225 Düsseldorf, Germany

**Keywords:** High-risk PCI, Acute kidney injury, Mechanical circulatory support, Impella, VA-ECMO

## Abstract

Acute kidney injury (AKI) is a common complication post-PCI. Here, in a single-center observational registry, we compared the frequency of AKI in patients at elevated risk for AKI (based on Mehran risk stratification scoring) who underwent VA-ECMO- or Impella-supported high-risk PCI. A total of 28 patients scheduled for elective high-risk PCI with mechanical circulatory support were studied prospectively. All patients were turned down for surgery due to exceedingly high risk. Allocation to VA-ECMO (n=11) or Impella (n=17) was performed according to site-specific restrictions on the daily availability of the VA-ECMO platform as a prospective enrollment and performed prior to initiation of PCI. We analyzed AKI incidence as our primary endpoint, as well as PCI success, duration, and peripheral complications. All patients were successfully revascularized and had MCS weaned at the end of the procedure. Baseline GFR and procedural contrast media were similar. Despite similar risks for AKI as calculated by the Mehran score (35 ± 18.9 vs. 31 ± 16.6 %; p=0.55), patients supported by Impella during PCI demonstrated a reduced incidence of AKI (55 vs. 12 %; p=0.03). MCS-assisted high-risk PCI with VA-ECMO or Impella is feasible. However, Impella is associated with a shorter procedure time and a lower incidence of AKI.

## Background

Acute kidney injury (AKI) is a common complication in patients treated by percutaneous coronary revascularization and is often linked to the pathophysiological impact of contrast media (CM) on the kidney [1, 4]. CM is the third leading cause of AKI in hospitalized patients [30, 42]. Patients who develop AKI have a 4- to 10-fold increase in mortality and significantly longer length of stay in the hospital, regardless of the severity of the disease [11]. AKI places a significant economic burden on both the patient and healthcare system [11]. Various studies have reported the periprocedural incidence of AKI in patients treated by primary percutaneous interventions (PCI) to range from 4 to 28%, depending on the definition of the AKI employed [12, 22, 33–35, 44]. In this patient population, AKI is strongly associated with in-hospital mortality, increased risk for myocardial infarction (MI), and dialysis [2, 3, 10, 14, 23]. There is no effective treatment for AKI after it develops, and current guidelines focus on preventative measures. The recently published PRESERVE trial results indicate that limiting patient exposure to CM is the best approach to preventing the development of AKI [45]. However, limiting CM is not always feasible [27]. New approaches to prevent AKI in high-risk patient populations are needed.

The risk of AKI is increased in MI patients where hemodynamics are compromised leading to depressed perfusion pressure and ultimately reduced kidney function [25]. Indeed, in an all-comers registry study, Tsai et al. reported a 7.1% rate of AKI in 985,787 patients undergoing PCI, compared to a 0.6–2.3% incidence rate in the general population [21, 44]. Several risk assessment tools, like the Mehran risk score or the Cigarroa quotient, have been developed with the aim of helping clinicians predict AKI after CM administration in order to provide patient-specific guidance [7, 16, 27].

Over the last decade, temporary mechanical circulatory support (MCS) devices have been increasingly used in the treatment of MI patients with complex coronary lesions and those at high risk for surgery [43]. These patients are severely hemodynamically compromised and often present with comorbidities like left ventricular ejection fraction less than 35%, NYHA heart failure class III/IV, diabetes mellitus, prior coronary artery bypass graft surgery, and/or a SYNTAX score greater than 30 [9, 34]. These comorbidities increase the patient’s risk for developing AKI. Limited data exists on how MCS support may affect the incidence of AKI in this patient population. While the use of the intra-aortic balloon pump (IABP) significantly increases the risk of AKI [5, 10], a recently published report demonstrated that hemodynamic support by the Impella microaxial pump actually protected high-risk patients from AKI despite the presence of underlying chronic kidney disease (CKD) [13]. These reports suggest that that device choice may differentially affect patient risk for developing AKI after high-risk PCI. However, no reports have compared this potential differential impact on AKI in this patient population. Understanding this impact may be a critical factor in how the best treatment option is chosen for these difficult to manage patients. Here, we examine how MCS support with the Impella and veno-arterial extracorporeal membrane oxygenation (VA-ECMO) differentially affects the development of AKI in patients undergoing scheduled high-risk PCI.

## Methods

### Patient Population

A total of 28 patients scheduled for elective MCS-supported high-risk PCI were included in the trial between September 2016 and September 2017. Emergency interventions (e.g., revascularization in ST-elevation myocardial infarction) were excluded from the trial. Typically, patients were diagnosed with complex coronary artery disease, severely reduced left ventricular ejection fraction, and/or various comorbid conditions in our unit or a tertiary referral hospital for which they were deemed suitable candidates for protected PCI or surgical revascularization. All patients were turned down for surgery due to exceedingly high risk as determined by the heart team decision. Allocation to VA-ECMO (n=11) or Impella (n=17) was performed according to site-specific restrictions on the daily availability of the VA-ECMO platform as a prospective enrollment.

All MCS-supported high-risk PCI were performed by the same four experienced interventionalists at the University Hospital in Dusseldorf. Arterial cannulation sites for Impella and VA-ECMO implantation were chosen by direct fluoroscopy using a crossover transfemoral approach. After needle puncture, two ProGlide systems (Abbott Vascular, Santa Clara, CA, USA) were inserted to facilitate efficient vascular closure at the end of the procedure. The general setup was very similar to a fully percutaneous TAVI procedure in the setting of sedation and local anesthesia. All interventionalists were skilled TAVI operators.

Prophylaxis of AKI was carried out according to current recommendations. In patients with an estimated GFR below 45 ml/min [8], a hydration protocol of delivering IV isotonic saline at 1 ml/kg body weight per hour for 12 hours prior to and 12 hours following the procedure was followed.

VA-ECMO support was initiated with the Cardiohelp system (Maquet Getinge Group, Rastatt, BW, D). Impella support was performed with the Impella 2.5 device (Abiomed, Danvers, MS, USA). Both MCS devices were set for hemodynamic support at 2.0 L/min during the procedure. The procedural time was defined as the time between the first puncture at the access site to the completion of the procedure at the point of closure.

AKI was defined according to the Kidney Disease International Global Outcomes Guidelines (KDIGO) by an increase in serum creatinine (SCr) ≥ 0.3 mg/dl within 48 to 72 h after contrast media exposure or an increase in SCr above 1.5 times from baseline. Incipient AKI was further classified, according to AKIN criteria graded for severity as *low* (max increase in SCr 0.3–0.59 mg/dL) with numerical value of 1, *moderate* (max increase in SCr 0.6–0.89 mg/dL) with a numerical value of 2, and *high* (max increase in SCr >0.9 mg/dL) with a numerical value of 3 [28]. Estimated GFR was calculated by the CKD-EPI equation. For reasons of clarity, the correct unit of ml/min/1.73m2 is presented in ml/min throughout the manuscript

### Risk Score Calculations

The Mehran risk scoring system utilizes a standardized risk calculator that includes eight variables. Each variable is assigned a weighted integer coefficient value to calculate a predicted AKI risk, expressed as both a risk score and percent risk of developing AKI based on the patient variables entered. AKI severity is then categorized into four groups: low-risk (risk score≤5), moderate-risk (6–10), high-risk (11–15), and very high risk (≥16). Accordingly, in the present study, the Mehran risk score for AKI, need for dialysis, and 1-year mortality were calculated for each patient in a post hoc analysis of collected patient data as previously described by Mehran and others (17, Flaherty MP, et al., Catheter Cardiovasc Interv. 2019).

### Statistical Analysis

Continuous variables are reported as mean ± SD. Discrete variables are reported as the percentage or categorical number. Statistical significance for continuous variables was determined by unpaired student *t* test. For discrete variables, statistical significance was determined by Fisher’s exact test. All statistical analysis was conducted on GraphPad Prism (GraphPad Software, La Jolla, CA). For all tests, a p <0.05 was considered to be significant.

## Results

### Baseline Clinical and Procedural Characteristics

There were no differences observed in the baseline clinical characteristics between the two groups (Table 1). There was a trend towards a reduced time of care within the intermediate and intensive care unit for the Impella group (3±3.0 vs. 8±7.1 days, p=0.072). Differences in procedural characteristics were observed (Table 2). Post-procedural hemoglobin was significantly lower in patients treated with VA-ECMO (9.1±1.1 vs. 10.6±2.1 mg/dL, p=0.034). Significantly more patients treated with ECMO required a transfusion of RBC (45% vs. 6%, p=0.022).

**Table 1:** Baseline patient characteristics

	Total (n=28)	VA-ECMO (n = 11)	Impella (n = 17)	p value
Clinical characteristics				
Age [years]	73 ± 9.9	73 ± 11.9	73 ± 8.8	0.979
Gender (male [%])	23 (82)	10 (91)	13 (77)	0.619
BMI [kg/m^2^]	27.1 ± 3.7	27.6 ± 4.5	26.9 ± 4.2	0.72
DM [%]	13 (46)	5 (45)	8 (47)	0.934
Insulin-dependent (yes [%])	8 (29)	3 (27)	5 (29)	0.624
COPD [%]	4 (14)	3 (27)	1 (6)	0.269
PAD [%]	5 (18)	4 (36)	1 (6)	0.062
Prior MI [%]	12 (43)	5 (45)	7 (41)	0.823
CABG [%]	4 (14)	0 (0)	4 (24)	0.132
Prior stroke [%]	4 (14)	3 (27)	1 (6)	0.269
2-vessel disease [%]	3 (11)	1 (9)	2 (12)	1.000
3-vessel-disease [%]	25 (89)	10 (91)	15 (88)	0.664
LVEF [%]	41 ± 16	39 ± 18	42 ± 14	0.618
NYHA I [%]	1 (4)	1 (9)	0 (0)	0.501
NYHA II [%]	5 (18)	2 (18)	3 (18)	0.671
NYHA III [%]	15 (54)	7 (64)	8 (47)	0.320
NYHA IV [%]	2 (7)	0 (0)	2 (12)	0.360
Valve disorders				
Mild and moderate aortic stenosis [numbers]	6 (21)	0 (0)	6 (35)	0.055
Mild and moderate mitral insufficiency [numbers]	20 (71)	10 (91)	10 (55)	0.099
Severe mitral insufficiency [numbers]	7 (25)	0 (0)	2 (12)	0.505
GFR at admission [ml/min]	70 ± 20	62 ± 18	75 ± 19	0.102
CKD I–II [%]	18 (64)	6 (55)	12 (71)	0.444
CKD III–IV [%]	12 (43)	5 (45)	5 (29)	0.444
Mehran risk for dialysis [%]	4.3±5.4	5.5±5.7	3.6±5.2	0.374
Mehran risk for 1-year mortality [%]	17.3±10.9	19.1±11.7	16.1±10.6	0.491
Hospital presentation				
STEMI [%]	0 (0)	0 (0)	0 (0)	1,000
NSTEMI [%]	12 (43)	4 (36)	8 (47)	0.705
UAP* [%]	6 (21)	2 (18)	4 (24)	0.736
Others [%]	8 (29)	3 (27)	5 (29)	0.903
Hospitalization [days]	12 ± 8.3	14 ± 10.9	10 ± 5.8	0.280
Standard care [days]	7 ± 4.2	6 ± 3.2	7 ± 4.8	0.613
Intermediate care unit (ICU [days])	3 ± 3.8	4 ± 4.3	2 ± 3.2	0.217
Intensive care unit (ICM [days])	2 ± 2.9	4 ± 4.0	1 ± 1.0	0.060
Intensive care (ICU + ICM [days])	5 ± 5.4	8 ± 7.1	3 ± 3.0	0.072

**Table 2:** Procedural characteristics

	Total (n=28)	VA-ECMO (n = 11)	Impella (n = 17)	p value
Procedural characteristics				
Radiation time [min]	32 ± 17	38 ± 20	29 ± 14	0.146
Lesions treated [number]	2 ± 0.8	2 ± 1.0	2 ± 0.7	0.407
DES implanted [number]	3 ± 2	3 ± 2	3 ± 1	0.427
Contrast medium [ml]	261 ± 106	293 ± 131	241 ± 84	0.258
Low osmolar contrast used (%)	28	11 (100)	17 (100)	1.000
Incidence of hemodialysis [%]	0 (0)	0 (0)	0 (0)	1.000
Laboratory samples				
Hb minimum post procedure [mg/dl]	9.9 ± 1.9	9.1± 1.1	10.6 ± 2.1	0.034
BNP (pg/ml)***	4783 ± 4541	6259 ± 5668	3834 ± 3556	0.219
Troponin at admission (ng/l)	175 ± 387	171 ± 345	177 ± 422	0.970
Troponin maximum post procedure (ng/l)	764 ± 2514	1463 ± 3967	312 ± 521	0.361
Complications				
Adverse events [%]	8 (29)	4 (36)	4 (24)	0.671
Vascular access site complications [%]	7 (25)	3 (27)	4 (24)	0.832
Bleeding [%]	5 (18)	3 (27)	2 (12)	0.353
Transfusion of red blood cells (RBC [%]	6 (21)	5 (45)	1 (6)	0.022
Transfusion of RBC [units]	1 ± 1.3	1 ± 1.6	0 ± 0.7	0.021
Pseudoaneurysm [%]	2 (7)	1 (9)	1 (6)	0.747
AV fistula [%]	1 (4)	0 (0)	1 (6)	0.413
Peripheral ischemia [%]	0 (0)	0 (0)	0 (0)	1.000
Dissection, thrombus, embolism [%]	0 (0)	0 (0)	0 (0)	1.000
CPR [%]	0 (0)	0 (0)	0 (0)	1.000
Vasopressors [%]	5 (18)	4 (36)	1 (6)	0.062
Peri-interventional stroke [%]	0 (0)	0 (0)	0 (0)	1.000

**Fig. 1:**
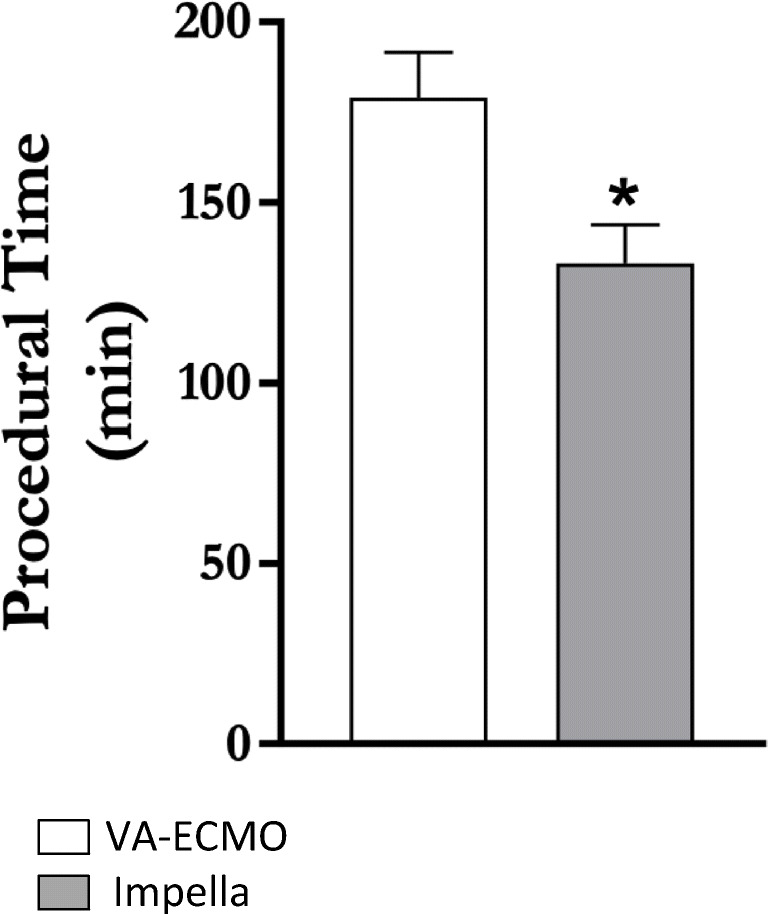
*Procedural characteristics*. The procedural time was shorter in the Impella cohort

**Fig. 2:**
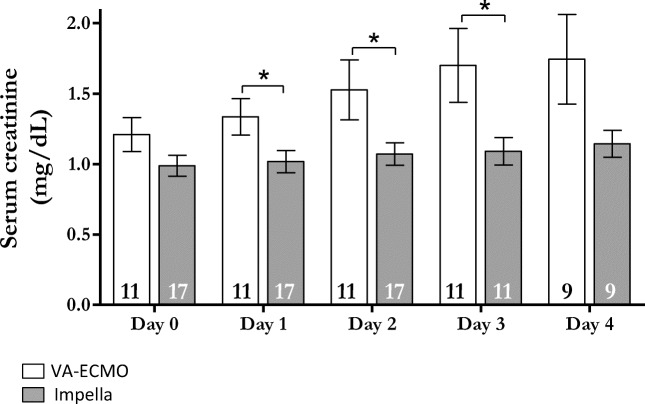
*Serial serum creatinine measurements following coronary revascularization.* SCr significantly increased in the VA-ECMO cohort compared to the Impella cohort at days 1–3 post-procedure. Numbers within the columns indicate the number of patients with creatinine measurements at each time point. The decrease in the number of patients at days 3 and 4 reflects the number of stable patients in which SCr measurements were no longer required

Figure 1 shows that procedural time was significantly shorter in Impella-supported patients (133±44.3 vs. 179±41.5 min, p=0.01), despite comparable complexity of coronary status as indicated by similar amounts of CM and coronary lesions treated (Table 2). Post-procedural SCr levels were significantly higher in patients supported with VA-ECMO only compared to those supported with Impella at day 1, day 2, and day 3. Post-procedural SCr levels in patients treated with Impella remained unchanged from day 0 through day 4 (Fig. 2).

### Incidence of AKI Is Lower in Impella-Supported Patients Despite a Similar Risk Score

**Fig. 3:**
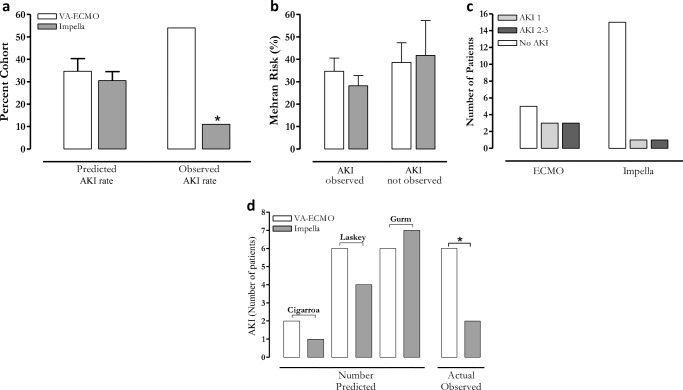
*Impella patients developed less AKI despite a similar risk.* **a** Right: the predicted rate of developing AKI in each cohort as calculated using the Mehran risk score (VA-ECMO = 35±19, Impella = 31±17%). Left: the actual observed rate of AKI (VA-ECMO = 54%; Impella = 12%). **b** The predicted rate of developing AKI using the Mehran risk score in subpopulations of patients who developed AKI (left, VA-ECMO=38±22%, Impella=42±22%, n.s.) and those who did not develop AKI (right, VA-ECMO=35±13%, Impella=28±18%, n.s.). These data indicate that that baseline risk developing AKI was similar in these patient subpopulations. **c** The rate of developing AKI 1 or AKI 2–3 was not different amongst the cohorts

Incidence of AKI is Lower in Impella-supported patients despite a similar risk score. The average risks for AKI in the VA-ECMO and Impella cohorts were similar (34.6±18.9% vs. 29.8±17.0%, p=0.49, Fig. 3**a**), respectively, as were the risk for NFD (5.4±5.7% vs. 3.6±5.2%, p=0.37, Table 1) and the risk for 1-year mortality (19.1±11.7% vs. 16.1±10.6%, respectively, p=0.49, Table 1). Despite a similar Mehran risk score for AKI, the actual observed rate of AKI in those patients supported by Impella was significantly lower compared to the observed rate of AKI in patients supported with VA-ECMO (12% vs. 54%, p=0.01, Fig. 3**a**). To determine if the risk was equally distributed between patients who developed AKI and those that did not, we assessed the calculated risk of these sub-cohorts independently (Fig. 3**b**). We found no differences in the predicted risk for AKI between these cohorts. Furthermore, the two groups did not vary regarding the severity of the resulting AKI (Fig. 3**c**). During follow-up, one patient in the VA-ECMO group developed CIN which progressed to end-stage renal disease with long-term dialysis.

Various protocols that assess risk for AKI are currently used, and there is no consensus gold standard. To account for any potential unintended bias in applying the Mehran risk score to our patient population, we assessed patient risk using three other published methods: the Cigarroa quotient, the Laskey risk calculation, and the Gurm risk calculation (Fig. 3**d**) [7, 15, 20]. Each of these risk assessment tools is binary predictors (yes/no) of increased risk for AKI and thus is applied discretely to each patient. Each of these risk assessment tools agreed with the Mehran risk score, demonstrating an equal risk of the respective patient populations for developing AKI.

### Incidence of AKI Is Independent of Baseline GFR and Volume of Procedural Contrast Medium

**Fig. 4:**
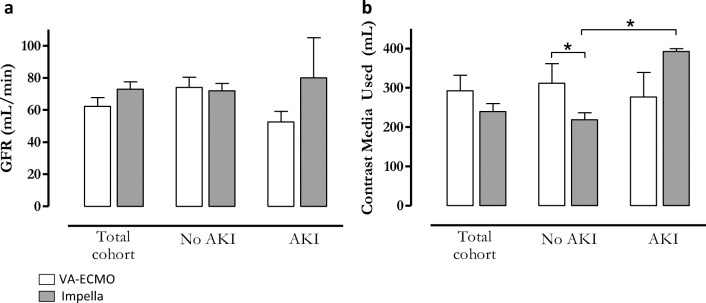
*The rate of AKI is independent of baseline GFR and amount of contrast media used*. **a** Baseline GFR did not differ when comparing entire cohorts (left), patients who did not develop AKI (middle), and patients who developed AKI (right). **b** The amount of contrast media used did not differ when comparing entire cohorts (left) or those patients who developed AKI (left). Impella patients who did not develop AKI received significantly less CM compared to patients supported with VA-ECMO who also did not develop AKI (middle). (n=11 VA-ECMO, 17 Impella, *p<0.05)

Acute kidney injury in high-risk PCI is linked to the reduction in glomerular filtration rate (GFR), renal hypoperfusion, and, in large part, to the amount of contrast used during the procedure (Flaherty, MP, Circ Res. 2017, Flaherty MP, CCI 2019, O'Neill WW. PROTECT II. Circ. 2012). Therefore, we examined whether any differences in these existed in our patient population. We found no differences in GFR at the time of admission either among the total cohorts or when comparing those who developed AKI and those who did not (Fig. 4**a**). The amount of contrast used did not differ among the cohorts. However, Impella patients who developed AKI received significantly more contrast compared to those who did not (218±69 vs. 392±11 mL, respectively, p=0.003). There were no differences in procedural contrast media amongst all the VA-ECMO groups.

### Post-Procedural Care

Although vascular access site bleeding was nearly the same in both groups (27 vs. 12 %; p=0.35, Table 2), the VA-ECMO group had significantly lower post-procedural hemoglobin (9.1±1.1 vs. 10.6±2.1 mg/dl, p=0.034, Table 2) and a higher rate of transfusion of red blood cells (45% vs. 6%, p=0.022, Table 2) compared to the Impella group. However, we believe this to be a dilution effect due to the required priming of the VA-ECMO system with 500 mL of saline. We observed similar decreases in both leucocytes and hematocrit, likely indicating a dilution-dependent effect.

## Discussion

The central finding of the current study is that the incidence of AKI among patients undergoing scheduled high-risk PCI was significantly higher in patients hemodynamically supported by VA-ECMO compared to those supported with Impella, despite the comparable calculated risk. This effect of Impella support was independent of baseline GFR and the amount of procedural contrast media [38, 39]. Our data suggest that the hemodynamic support supplied by the Impella pump differentially affects patient outcomes post high-risk PCI. Prior to this study, in our center, clinical equipoise on the particular advantages of VA-ECMO or Impella support during high-risk PCI existed; hence, the choice of hemodynamic support was left to the discretion of the clinical operator. However, the data presented here indicate that Impella support may procure kidney function during protected PCI in contrast to VA-ECMO. For this reason, the implementation of VA-ECMO in the context of protected PCI is now limited to very few indications (e.g., severe right heart failure) in our center.

In line with previous data by Flaherty MP et al., regarding predictors of AKI in HR-PCI populations, AKI incidence in Impella-supported patients in our study was independent of baseline eGFR and the amount of procedural contrast media (Flaherty MP, Circ Res, and Flaherty MP CCI. 2019). However, these authors reported disparate findings in terms of AKI predictors during HR-PCI between the two studies: one in a control-matched cohort and the other using patients from the Global cVAD study. Using multivariate logistic analysis, these authors demonstrated that when contrast volumes exceeded 280 cc (median contrast used 287: IQR, 225-320) versus 180 cc (180: IQR, 125.0-250.0), contrast acts as a predictor of AKI. Here, we report comparable contrast volumes (293 ±131, ECMO; 241 ± 84, Impella), yet the incidence of AKI was elevated in the VA-ECMO group. In addition, these authors reported that baseline SCr predicted AKI in one study yet failed to do so in a similar population from the cVAD Study. The authors concluded that this incongruence was likely explained by the significantly higher baseline SCr in the cVAD population compared to a control-matched cohort (average Cr, 1.53±0.13 vs. 1.26±0.24 mg/dL).

Our study suggests important differences exist between the hemodynamic support supplied by VA-ECMO compared to the Impella CP that result in a variable incidence of AKI. While we observed procedural differences between our VA-ECMO and Impella cohorts like shorter procedural times in the Impella group, we believe this difference do not account for the higher incidence of AKI in patients support with VA-ECMO. Furthermore, VA-ECMO and Impella support increase mean arterial pressure to a similar extent, suggesting that the observed difference is likely not related strictly to perfusion pressure. Existing data indicate key differences between ECMO and Impella support that may mediate this effect either by themselves or (more likely) in combination with one another: hemodynamic considerations, effects atrial natriuretic peptide (ANP) production, and a differential inflammatory response.

VA-ECMO patients exhibited lower hemoglobin values and a higher need for blood transfusions during postinterventional care. Thus, CM-induced kidney injury may have been aggravated in the VA-ECMO cohort by an additional prerenal component caused by peri-interventional blood losses related to the more complex cannulation procedures also mirrored by prolonged procedural time. In this trial, we noted acceptable rates of peripheral vascular complications limited to bleeding, AV-fistula formation, and pseudoaneurysms without the need for surgical or interventional revascularization. It has been discussed that the potential benefits of MCS may be devastated by detrimental side effects of provoked vascular complications (Schrage, Circulation 2020). Thus, we recommend to weigh the risks/benefits of mechanical circulatory support before assuming liberal use of these platforms and limit its implementation to interventionalists trained in large-bore access site management.

The importance of pulsatile flow in maintaining renal cortical blood flow and kidney function has been demonstrated [31, 40, 41]. VA-ECMO is a continuous flow device that results in non-pulsatile (or low pulsatile) blood flow. The placement of the inflow and outflow cannulas in the femoral vein and artery, respectively, combined with the power of the pump in this device increases mean arterial pressure and dampens the contribution that the native heart provides to forward flow. The result is a narrowing or even absence of pulse pressure [6]. This would be expected to be more pronounced in patients with pre-existing hemodynamic compromise such as the high-risk population where native heart function is already impaired. The Impella is also a continuous flow device, but it varies from ECMO in two critical ways. First, this device is placed across the aortic valve within the inflow cage in the left ventricle and the outflow cage in the ascending aorta. Second, the maximum flow of the Impella CP is lower than ECMO; thus, it is unable to completely supplant the contribution of the native heart to forward flow. Flow-through the Impella cannula is subject to the pressure changes of the contracting ventricle, and pulsatile flow is maintained, while the mean arterial pressure is augmented. The continuous non-pulsatile flow of ECMO has been linked to the redistribution of intrarenal regional blood flow, resulting in poor kidney function [40]. This difference between ECMO and Impella to maintain pulsatile flow likely plays an important role in the effect we observe here. More detailed research is required to investigate this potential.

Second, both devices increase perfusion pressure to a similar extent, but the devices have fundamentally different effects on left ventricular preload and afterload. This may potentially alter downstream ANP production. ANP is produced by atrial myocytes in response to myocardial stretch resulting from volume and pressure overload. In an effort to decrease blood volume and relieve atrial stretch, ANP release increases sodium and water excretion. Important to this study, ANP also inhibits the renin-angiotensin-aldosterone system (RAAS). RAAS-dependent signaling is a principal player in preventing low GFR.

In the setting of VA-ECMO support, large increases in afterload impair the ability of the left ventricle to eject blood sufficiently. This leads to congestion and distention of the left ventricle and atrium. This ECMO-dependent effect on afterload is known to require venting in order to relieve the resulting wall stress [26, 36]. Reflecting this atrial stretch, serum ANP is increased after VA-ECMO treatment [32, 37]. Normally, ANP signaling ceases upon the alleviation of atrial stretch (resulting from natriuresis). Yet, in the setting of VA-ECMO, afterload is artificially preserved leading to elevated atrial pressure. This would maintain ANP-dependent signaling and inhibition of the RAAS. In this respect, Impella support is fundamentally different. Due to its placement, the Impella directly unloads the left ventricle, and upstream unloading of the left atrium is observed [17]. Therefore, atrial stretch is less developed under Impella support, and ANP/RAAS signaling would be normalized. This important distinction between these devices is already exploited in the clinical setting. Currently, VA-ECMO patients presenting in cardiogenic shock are often vented by inserting the Impella device to mechanically unload the heart while on VA-ECMO support [26].

Lastly, ECMO is known to induce a strong inflammatory response which can inhibit kidney function [19, 24]. The inflammatory response is observed during both the acute (<2 h) and long-term (>48 h) support [18]. It is believed this response is caused by prolonged blood exposure to foreign surfaces within the membrane oxygenator of the VA-ECMO circuit. Currently, there are insufficient data on the inflammatory response induced by the Impella to be conclusive. However, emerging data suggest that Impella support may suppress inflammatory signaling in supported hearts [29].

While small, our study indicates that the choice between VA-ECMO or Impella support during high-risk PCI procedures may have important implications in terms of the rate of AKI. Our data suggests that the inclusion of Impella support into current risk assessment tools may be warranted, although a larger clinical study would be required to confirm this.

## Limitations

Several limitations should be mentioned. (1) The trial is of a small sample size thus hampering any conclusions beyond the incidence of AKI. Our trial deciphered differential short-term effects on kidney function by Impella and VA-ECMO. It was not designed to analyze the long-term outcome, which is certainly of utmost importance for the potential clinical benefit. However, it is the first trial to compare VA-ECMO- and Impella-assisted PCI in a balanced cohort where all indications and interventions were undertaken by a small group of interventionalists. Due to a small sample size, we cannot decipher how the trend of elevated vasopressor use during VA-ECMO-assisted PCI relates to the incidence of AKI. (2) The Mehran score is not a dedicated score to predict AKI in the setting of MCS-protected PCI. Nevertheless, we chose the Mehran score to characterize AKI risk since it has been derived from a large all-comers cohort including patients with advanced heart failure and various comorbidities that are embedded in the score. (3) Despite careful patient characterization, we lack data of right-heart catheterizations. Thus, conclusions on right-heart congestion that are known to play a central role in the development of AKI in the context of heart failure cannot be made.

## Conclusion

MCS-assisted high-risk PCI with VA-ECMO or Impella is feasible. However, implementation of Impella is associated with a shorter procedure time and a lower incidence of AKI. This study highlights the importance of device choice in hemodynamically compromised patients undergoing PCI. While both the Impella and VA-ECMO can suitably provide hemodynamic support, the secondary effects of the devices have a direct impact on patient outcomes.

## Abbreviations

AKI: Acute kidney injury
ANP: Atrial natriuretic peptide
CKD: Chronic kidney disease
CM: Contrast media
GFR: Glomerular filtration rate
HR-PCI: High-risk percutaneous coronary intervention
IABP: Intra-aortic balloon pump
MCS: Mechanical circulatory support
MI: Myocardial infarction
PCI: Percutaneous coronary intervention
SCr: Serum creatinine
VA-ECMO: Veno-arterial extracorporeal membrane oxygenation
